# Chitosan Hydrogel-Delivered ABE8e Corrects PAX9 Mutant in Dental Pulp Stem Cells

**DOI:** 10.3390/gels9060436

**Published:** 2023-05-25

**Authors:** Bowen Liu, Chenjiao Zhang, Han Zhao, Jian Gao, Jingchao Hu

**Affiliations:** 1 Outpatient Department of Oral and Maxillofacial Surgery, Beijing Stomatological Hospital, School of Stomatology, Capital Medical University, Tian Tan Xi Li No. 4, Beijing 100050, China; bowen_dent@163.com; 2 Department of General, Beijing Stomatological Hospital, School of Stomatology, Capital Medical University, Tian Tan Xi Li No. 4, Beijing 100050, China; chenjiaozhang@foxmail.com; 3 Multi-Disciplinary Treatment Center, Beijing Stomatological Hospital, School of Stomatology, Capital Medical University, Tian Tan Xi Li No. 4, Beijing 100050, China; z75301704@163.com; 4 State Key Laboratory of Oncogenes and Related Genes, Shanghai Cancer Institute, Shanghai Jiao Tong University School of Medicine, Shanghai 200011, China; 5 Department of Periodontics, Beijing Stomatological Hospital, School of Stomatology, Capital Medical University, Tian Tan Xi Li No. 4, Beijing 100050, China

**Keywords:** hydrogel, hypodontia, dental agenesis, dental pulp stem cells, PAX9, ABE8e

## Abstract

Hypodontia (dental agenesis) is a genetic disorder, and it has been identified that the mutation C175T in PAX9 could lead to hypodontia. Cas9 nickase (nCas9)-mediated homology-directed repair (HDR) and base editing were used for the correction of this mutated point. This study aimed to investigate the effect of HDR and the base editor ABE8e in editing PAX9 mutant. It was found that the chitosan hydrogel was efficient in delivering naked DNA into dental pulp stem cells (DPSCs). To explore the influence of the C175T mutation in PAX9 on the proliferation of DPSCs, hydrogel was employed to deliver PAX9 mutant vector into DPSCs, finding that the PAX9-containing C175T mutation failed to promote the proliferation of DPSCs. Firstly, DPSCs stably carrying PAX9 mutant were constructed. Either an HDR or ABE8e system was delivered into the above-mentioned stable DPSCs, and then the correction efficiency using Sanger sequencing and Western blotting was determined. Meanwhile, the ABE8e presented significantly higher efficiency in correcting C175T compared with HDR. Furthermore, the corrected PAX9 presented enhanced viability and differentiation capacity for osteogenic and neurogenic lineages; the corrected PAX9 even possessed extremely enhanced transcriptional activation ability. In summary, this study has powerful implications for studies into base editors, chitosan hydrogel, and DPSCs in treating hypodontia.

## 1. Introduction

Hypodontia (dental agenesis) is a condition of tooth loss, and the agenesis of permanent teeth is a clinically challenging problem in humans. Hypodontia describes the absence of one to six teeth, excluding third molars, and the global incidence is 1.6–9.6% [1]. Tooth development is under strict genetic control, and paired box 9 (PAX9) is a transcription factor that is expressed in the mesenchyme during the early stages of tooth development [2,3,4,5]. The PAX9 gene is located in chromosome 14, and PAX9 mutations are related to congenital tooth missing cases. Among PAX9 mutations, the heterozygote transition of C175 to T creates a stop codon at arginine 59 (Arg59) in the protein, which is located in the DNA-binding pair box of exon 2 [6,7]. Therefore, this mutation in PAX9 causes dental hypoplasia.

Human dental-pulp-derived stem cells (DPSCs) are mesenchymal stem cells with plastic adhesion and fibroblast-like morphology. Studies have shown that they express specific markers CD90 and CD73 of mesenchymal stem cells [8]. DPSCs are a highly heterogeneous population with different cloning and expression markers, as well as differences in proliferation and differentiation abilities [9]. DPSCs can differentiate into various lineages such as odontoblasts, adipocytes, and neurons in vitro [10].

Correcting mutated genes in stem cells is an attractive therapy for hereditary dis-eases which requires the delivery of gene repair tools to these cells [11,12]. To a large extent benefitting from nanocarriers, nonviral vector systems are promising alternative to virus-based gene delivery counterparts [13]. The nanocomplex revealed good biocompatibility and transfection efficiency. It has been found that hydrogel could also enhance vector retention and increase gene delivery [14,15].

Hydrogel is a three-dimensional (3D) cross-linked molecular network established by a covalent bond or noncovalent interaction. The network’s noncovalent interaction mainly includes physical entanglement, hydrogen bonds, hydrophobic interaction, coordination interaction, and so on [16]. Many properties of hydrogel, such as strength, toughness, and adhesive ability, could be well controlled to realize its application in tissue engineering, biomedicine, biosensors, and other fields. Furthermore, hydrogels can hold a large amount of water. Therefore, researchers try to synthesize hydrogels for studying a series of natural polymers and synthetic polymers [17]. By introducing glycerol or mixed nanoparticles into the hydrogel network, excellent self-healing properties and adhesion can be obtained [18]. Therefore, hydrogel provides an interesting and ambitious approach to deliver PAX9 modification systems into stem cells.

Because of their superior properties, hydrogels are widely used in many fields. The rich water and 3D network structure of hydrogels make them have strong diffusivity and provide a physiological simulation environment for cell/tissue metabolism, which has excellent prospects in biomedicine, such as drug delivery, cell culture, tissue regeneration, etc. [18]. Hydrogel could be applicated in cartilage tissue regeneration, corneal defect healing [19], and wound healing acceleration [20]. A hydrogel with stable mechanical properties and hemostatic activity is used for tissue adhesion [21]. A conductive self-healing hydrogel with injectability, strain/motion sensing capability, and its application in nerve regeneration has been proved [22]. Furthermore, hydrogel could also be used in DPSCs research. A shear-thinning, ROS-scavenging hydrogel has been combined with DPSCs to promote spinal cord repair [23]. It was also reported that multicellular spheroids formation on hydrogel could enhance osteogenic/odontogenic differentiation of DPSCs under magnetic nanoparticles induction [24].

Chitosan, the deacetylated form of chitin, is used in a broad range of applications in biomedicine because it has proved to be nontoxic, biocompatible, and biodegradable [25,26,27]. Because of the active amino groups and hydroxyl groups in its polycationic structure, chitosan is easy to modify. It can effectively bind negatively charged domains, such as drugs, DNA, and RNA, to transport these substances into cells [28,29,30]. Positively charged chitosan can bind to a negatively charged CRISPR/Cas9 system through electrostatic interactions, forming stable peptides [30]. In addition, due to the negative charge on the cell and nuclear membranes, the interaction with positively charged chitosan leads to the absorption of transporters and nuclear migration [31].

The clustered regularly interspaced short palindromic repeats/Cas9 nuclease (CRISPR/Cas9) system is a widely accepted and efficient gene-editing technology [32,33]. Some studies used chitosan to transport CRISPR/Cas9 delivery systems to cells for genome editing [34]. For example, it was reported that chitosan nanoparticles were employed to load the CRISPR/Cas9 system and transport it to fibroblasts for editing the gene BMPR2 that causes hereditary pulmonary hypertension (PAH) [35]. In another study, chitosan was used to coat nanoparticles that were based on polylactic acid glycolic acid (PLGA). It transported the CRISPR/Cas9 system to HEK-293 cells, which inhibited the expression of green fluorescent protein (GFP) by up to 80% [36]. There are currently a variety of Cas9-based technologies in use, such as homology-directed repair (HDR) and the adenine base editors (ABEs) for the treatment of genetic diseases [37,38]. The double nicking by gRNA pair-guided Cas9 nickase (nCas9) reduces off-target mutagenesis by 50- to 1000-fold compared with wild Cas9 [39]. On the other hand, Liu et al. assembled nCas9 and the adenine deaminase TadA into ABE8e, which could convert A•T to G•C [40,41,42]. Furthermore, the catalytic deamination effect of advanced ABE8e increased more than 1000-fold compared with its ABE counterparts [43].

To explore the application of gene editing in the treatment of tooth agenesis, it was first proved that the PAX9 mutant failed to promote the proliferation of dental pulp stem cells (DPSCs). Then, the in vitro PAX9 mutant models were generated based on the C175T mutation for the exploration of gene correction. The ABE8e demonstrated substantially improved editing efficiencies compared with HDR. Furthermore, hydrogel enhanced the delivery of ABE8e into DPSCs and finally raised the correction of PAX9 mutant and improved the proliferation and differentiation of DPSCs. This study provides an innovative approach for gene therapy by employing nanoparticles to deliver the advanced gene editor ABE8e to stem cells.

## 2. Results

### 2.1. Preparation of Hydrogel Based on Chitosan

**Figure 1:**
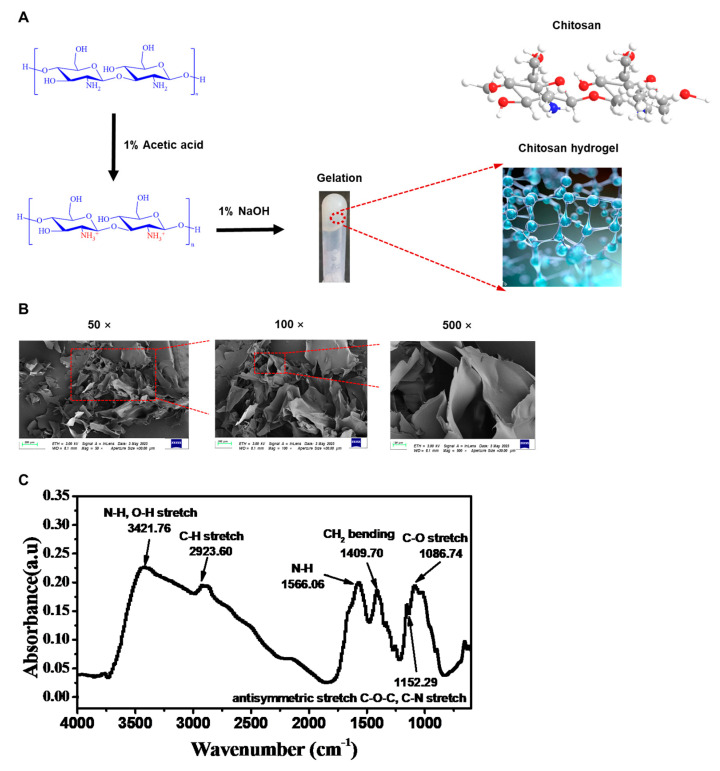
Physicochemical properties of chitosan hydrogel: (**A**) The preparation of chitosan-based hydrogel. SEM images of the chitosan-based hydrogel in (**B**) 50×, 100×, and 500×. (**C**) FTIR spectra of the chitosan-based hydrogel.

To transport the CRISPR/Cas9 system into DPSCs using chitosan hydrogel in this study, chitosan hydrogel was first synthesized (Figure 1A). The deacetylated chitosan solution was prepared by dissolving it in acetic acid solution at room temperature. The pH of the deacetylated chitosan solution was adjusted to neutral and it was refrigerated. After dialysis for 1–3 days, the hydrogel was centrifuged to remove the unconjugated water to prepare the chitosan hydrogel. SEM was used to evaluate the surface morphology, pore morphology, and pore size distribution of the chitosan hydrogel (Figure 1B). The chemical structures of chitosan hydrogel were determined by FTIR (Figure 1C). Compared with chitosan reported in the previous literature [44], it is obvious that the characteristic peaks (O-H) in the chitosan hydrogel shifted from 3290 cm^−1^ and became broad after the chitosan hydrogel formation. The shift in partial IR absorption bands (e.g., amide bond and CH_2_ bending) to a low wave number—the peaks shifted from 1584 to 1566.06 cm^−1^ and from 1414 to 1409.7 cm^−1^—suggest hydrogen bonding among amino groups and hydroxyl groups, which are exposed on the chitosan molecular chain.

### 2.2. Hydrogel Improved Plasmid Delivery into DPSCs

**Figure 2:**
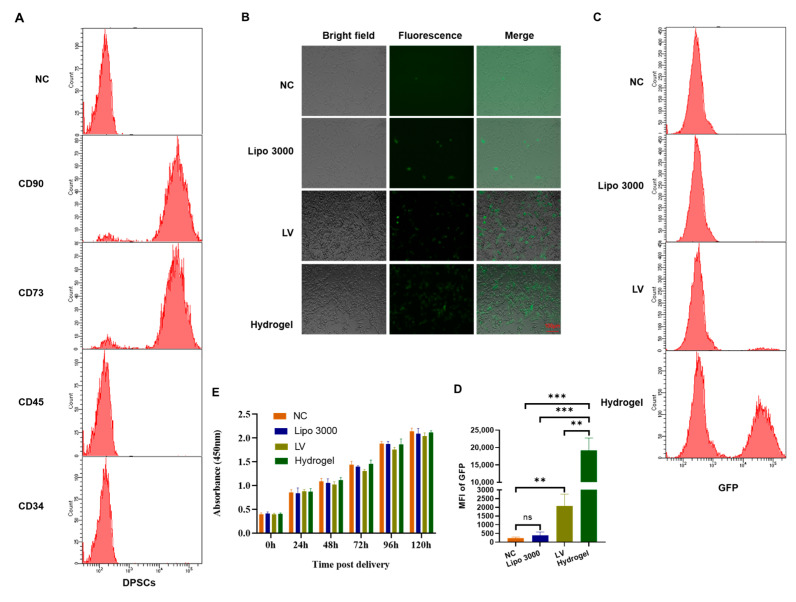
The influence of hydrogel on the delivery of DNA to DPSCs: (**A**) Flow cytometry assessment of mesenchymal markers CD90 and CD73 and hematopoietic markers CD45 and CD34 on DPSCs. GFP expression plasmids were delivered using Lipofectamine™ 3000 or hydrogel. (**B**) The fluorescence was measured using microscope. NC, negative control, no treatment. LV, lentiviral vector expressing GFP. (**C**) Representative histogram of flow cytometry assessment for the measurement of fluorescence intensity. (**D**) Mean fluorescence index of fluorescence intensity from flow cytometry assessment. (**E**) CCK-8 assays for evaluating the influence of hydrogel on DPSCs. The assay was conducted at 24, 48, 96, and 120 h post delivery. All data are from experiments performed in triplicates; ns, not significant; ** *p* < 0.01, *** *p* < 0.001.

Firstly, the DPSCs characteristics were confirmed through flow cytometry assessment. The DPSCs were positive for the mesenchymal markers CD90 and CD73 and negative for the hematopoietic markers CD45 and CD34 (Figure 2A). It was reported that transfection of DPSCs using lipofectamine is poor [45], and nanoparticles have potential to improve transfection of stem cells. The influence of Lipofectamine™ 3000, lentiviral vector, and chitosan hydrogel on delivering the GFP-expressing vector into DPSCs was first explored. Through the detection of fluorescence using microscope (Figure 2B) and flow cytometry assessment (Figure 2C,D), it was found that hydrogel could markedly improve the transfection. Then, Cell Counting Kit-8 (CCK-8) assay was utilized to detect the influence of hydrogel on cell viability, finding that the polypeptides presented mild influence on DPSCs viability (Figure 2E).

### 2.3. C175T Mutation Eliminates the Role of PAX9 in Promoting DPSCs Proliferation

**Figure 3:**
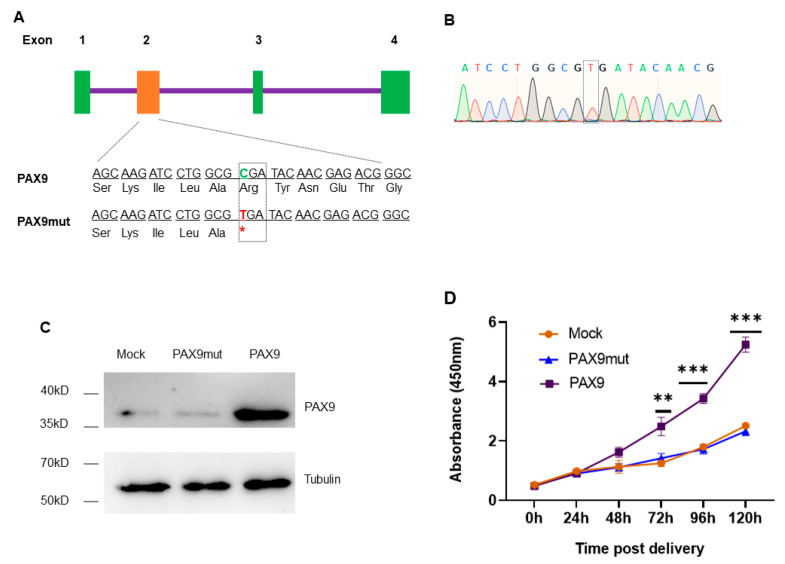
The influence of C175T in PAX9 on the proliferation of DPSCs: (**A**) Construction of wild-type and mutated PAX9 plasmids. The mutated PAX9 contains the point mutation C175T. *, stop codon. Green rectangle indicates Exon 1, 3, 4. Orange rectangle indicates Exon 2. (**B**) Sequence of PAX9mut in vector. Green oscillogram indicates base A. Red oscillogram indicates base T. Blue oscillogram indicates base C. Black oscillogram indicates base G. (**C**) Western blotting assays for evaluating the expression of PAX9 in DPSCs. Mock vectors, PAX9mut vectors, and PAX9 vectors were delivered to DPSCs using hydrogel. (**D**) CCK-8 assays for evaluating the influence of C175T in PAX9 on the proliferation of DPSCs. Error bars represent mean ± SD (*n* = 3). ** *p* < 0.001, *** *p* < 0.0001.

PAX9 is expressed in the mesenchyme during tooth development; the point mutation of C175T creates a stop codon in the original position of Arg59, meaning early termination of translation [4]. According to the coding sequence (CDS) of PAX9, the vector carrying the PAX9 CDS with C175T mutation (Figure 3A,B, Scheme S1A) was constructed. To evaluate the function of PAX9, the chitosan hydrogel was used to deliver the vector carrying the wild or mutated PAX9 CDS into DPSCs. Seventy-two hours post delivery, the total cellular protein was extracted, and the protein level of PAX9 was measured using Western blotting. The results show that the wild PAX9 vector overexpressed PAX9 in DPSCs, while the mutated PAX9 vector failed to express PAX9 in DPSCs (Figure 3B, Scheme S1B). Then CCK-8 assay was conducted to detect the influence of modified PAX9 on the proliferation of DPSCs, finding that mutated PAX9 had no effect on the promotion of DPSCs proliferation (Figure 3C).

### 2.4. Correction of Mutated PAX9 Using nCas9-Mediated HDR

**Figure 4:**
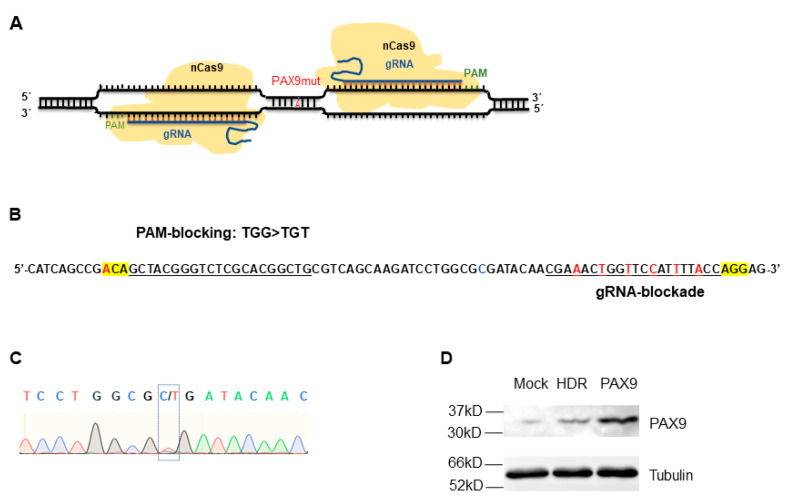
Modification of PAX9 mutant using HDR: (**A**) Schematic of PAM-out gRNA design for HDR. (**B**) Schematic of PAM blocking and gRNA blocking in HDR template. The two PAMs are highlighted in yellow. The target sequence of gRNA (upper) and gRNA (lower) are underlined. The mutation for PAM blocking (upper) and gRNA blocking are in red. (**C**) Sanger sequencing of PAX9 72 h after HDR delivery into DPSCs by hydrogel. Green oscillogram indicates base A. Red oscillogram indicates base T. Blue oscillogram indicates base C. Black oscillogram indicates base G. (**D**) Western blotting assays for evaluating the expression of PAX9 72 h after HDR delivery into DPSCs by hydrogel in DPSCs.

To correct the C175T mutation in PAX9, chitosan hydrogel was used to deliver the HDR correction system into DPSCs stably carrying PAX9 with the C175T mutation. According to the position of the C175T mutation, theoretically, it is suitable for repair by HDR and ABE8e. Then, the PAM-out gRNA pairs for repairing PAX9 mutant using HDR were designed (Figure 4A). To increase HDR accuracy, PAM blocking and gRNA blocking in the HDR template were introduced through synonymous mutation. The PAM blocking was TGG > TGA at the end of one gRNA, and the gRNA blocking was the mutation of another gRNA sequence in the HDR template (Figure 4B). PAX9 was sequenced and determined the protein level of PAX9 72 h post delivery of HDR; the Sanger sequencing showed that HDR corrected the C175T mutation to some extent (Figure 4C). To further validate the correction efficiency, the PAX9 protein levels were detected using Western blotting, finding that PAX9 protein level increased mildly (Figure 4D). The results indicate that HDR could correct the C175T mutation, but it is not very efficient; so, to improve the correction efficiency, it is necessary to find more effective solutions.

### 2.5. Correction of Mutated PAX9 Using Cas9-Mediated ABE8e

**Figure 5:**
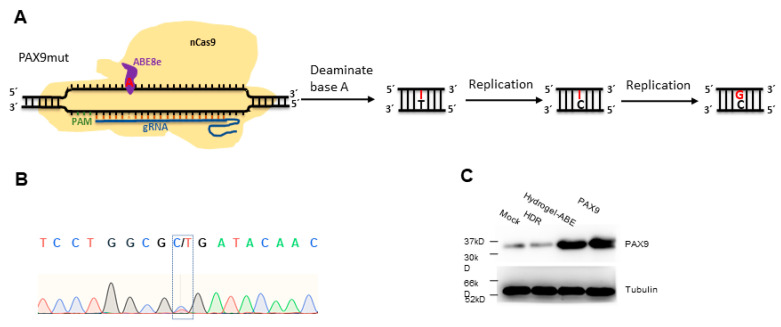
Modification of PAX9 mutant using ABE8e: (**A**) Schematic of the ABE8e-mediated base editing for PAX9 mutant (PAX9mut). (**B**) Sanger sequencing of PAX9 72 h after ABE8e delivery into DPSCs by hydrogel. Green oscillogram indicates base A. Red oscillogram indicates base T. Blue oscillogram indicates base C. Black oscillogram indicates base G. (**C**) Western blotting assays for evaluating the expression of PAX9 72 h after ABE8e delivery into DPSCs by hydrogel.

Owing to the high efficiency of ABE8e in correcting base A to G in the DNA context [40], chitosan hydrogel was also employed to deliver ABE8e to DPSCs stably carrying PAX9 with the C175T mutation (Figure 5A). Seventy-two hours post delivery of ABE8e system, PAX9 was sequenced, and the Sanger sequencing showed that ABE8e corrected the C175T mutation remarkably (Figure 5B). In addition, to explore the correction efficiency of ABE8e, the protein level of PAX9 was determined 72 h post delivery of HDR or ABE8e system using Western blotting. The results show that ABE8e presented more efficient correction compared with HDR (Figure 5C). The results indicate that ABE8e mediated significantly more efficient correction of C175T compared with HDR.

### 2.6. Hydrogel-Delivered ABE8e Improves the Proliferation and Differentiation of DPSCs

**Figure 6:**
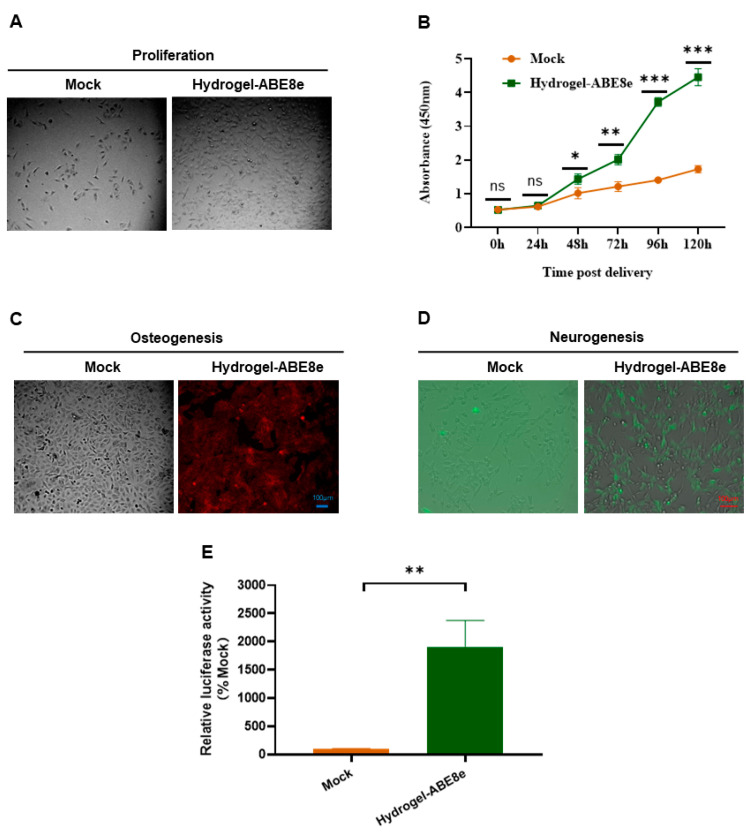
Impact of hydrogel-delivered ABE8e on DPSCs functions: (**A**) Photos of proliferated DPSCs. (**B**) CCK-8 assays for evaluating the influence of modified PAX9 on DPSCs proliferation. The assay was conducted at 24, 48, 96, and 120 h post delivery. All data are from experiments performed in triplicates. (**C**) Alizarin red staining after osteogenic induction for 21 days. (**D**) Immunofluorescence staining after neurogenic induction for 7 days (green: TuJ1). (**E**) Dual luciferase assays for the evaluation of the influence of PAX9 on the transactivation of the BMP4 promoter. Error bars represent mean ± SD (n = 3); ns, not significant; * *p* < 0.05, ** *p* < 0.01, *** *p* < 0.001.

Chitosan hydrogel was used to deliver the ABE8e editing system into DPSCs. Seventy-two hours post plasmid delivery, the cells were reseeded and photographed forty-eight hours later, showing that the ABE8e correction group showed more cells compared with DPSC delivered with mock plasmids (Figure 6A). Furthermore, the CCK-8 assay showed that hydrogel-ABE8e-modified DPSCs showed higher viability (Figure 6B). Furthermore, hydrogel-ABE8e-modified DPSCs demonstrated improved differentiation capacity into osteogenic (Figure 6C) and neurogenic lineages (Figure 6D). Considering that the BMP4 promoter is the downstream transcription target of PAX9 [46], the dual luciferase assay was conducted to test the transactivation ability of PAX9. The gene-editing vectors with BMP4 promoter–reporter construct were cotransfected into the DPSCs. The results of relative luciferase activity show that the modified PAX9 by ABE8e possessed extremely enhanced transcriptional activation ability (Figure 6E).

## 3. Discussion

Tooth development is strictly genetically regulated, and PAX9 mutations could cause dental hypodontia. In the PAX9 mutation, the heterozygous transition from C175 to T produces a stop codon at arginine 59 (Arg59) in the protein [4]. This mutation prevents the translation of PAX9 and attenuates its ability to activate transcription, thereby causing missing teeth.

The delivery of gene therapy tools to stem cells via nonviral vectors would be an at-tractive alternative to the treatment of hypodontia. This strategy eliminates the risk of multiple viral vectors mentioned above [47,48]. It was recently reported that gene-editing tools could mediate efficient base editing in primary and immortalized cells [49], so it was tried in immortalized stem cells. However, the transfection efficiency of stem cells is very low. According to reports, hydrogel could improve transfection efficiency and therefore serves as a particularly potential DNA delivery vector [15].

Hydrogels are highly hydrophilic three-dimensional network gels formed by hydrophilic polymers which have been studied for more than 100 years [50]. Hydrogels can be divided into natural hydrogels and synthetic hydrogels. One component of water gel can obtain more robust physical or chemical characteristics by combining different raw materials. In addition, hydrogels have different sizes (microgel, nanogel, etc.). With the progress of technology, self-healing injectable hydrogels, robust viscous hydrogels, and even conductive hydrogels for nerve injury repair have entered the research stage. Researchers are trying to use various drugs and bioactive factors to enhance the function of hydrogels. Developing hydrogels with higher biological activity and function is of great significance. Some medicines are injected into the desired position in the solution state and then become a gel, which allows the drug to be administered with minimal invasion and eliminates the need for surgery to implant prefabricated materials. The network has good biocompatibility [51,52], swelling [53], and mechanical characteristics similar to the soft tissue extracellular matrix [54], making it widely used in tissue engineering, drug, and gene delivery, as well as cell morphogenesis [55,56,57]. Hydrogel has the characteristics of easy production, biocompatibility, and large cargo capacity [58,59]. Therefore, hydrogel is very advantageous for us to safely transport gene therapy systems with large molecular weight. In our study, the GFP expression plasmid delivery indicated that hydrogel enhanced plasmid transfection into DPSCs.

Human dental pulp mesenchymal stem cells (hDPMSCs) are composed of a variety of subtypes, including DPSCs, periodontal ligament stem cells (PDLSCs), apical papillary cells (SCAP), and alveolar bone marrow stem cells (ABMSCs), obtained from different types of dental tissue [7]. Gronthos et al. isolated and identified DPSCs for the first time. They have powerful proliferative properties and can differentiate into bone [60], dentin [61], and nerve [62], with high angiogenic and neurogenic potential. In addition, preclinical/clinical studies have demonstrated the therapeutic potential of DPSCs for the regeneration of various tissue diseases, including ischemic brain injury, infarcted myocardium, muscular dystrophy, and dental caries [63].

In this research, DPSCs stably carrying PAX9 with the C175T mutation were established. The dental pulp is essential for maintaining teeth, providing nutrition and oxygen supply, innervation, reactive/restorative dentin formation, and immune response [64]. Caries not only destroy the rigid structure of teeth but also cause pulp necrosis in severe cases. Current dental pulp treatment schemes cannot achieve pulp regeneration. The most common method for treating pulp necrosis is to remove damaged pulp tissue, which can lead to loss of tooth vitality and increase tooth fragility. However, DPSCs isolated from dental pulp tissue exhibit mesenchymal stem-cell-like characteristics and are fibroblast-like adherent cells characterized by colony-forming activity, self-renewal, and multiple lineage differentiation potentials. Like other mesenchymal stem cells, DPSCs have received considerable attention in regenerating damaged dental pulp tissue due to their pluripotency, high fertility, and viability after cryopreservation [65]. DPSC can be easily isolated from discarded teeth and stored in stem cell banks for a long time, improving the convenience and operability of its application [66]. DPSC has a high proliferation and migration ability and the immune regulation and angiogenesis/neurotrophic effects of secretors [67]. DPSC is nonimmunogenic and does not trigger an allogeneic immune response [68]. Several studies have isolated highly proliferative dental pulp cells from adult dental pulp tissue and differentiated them into odontoblasts and mineralization in vitro [69].

The regeneration of dental pulp tissue is essential in dentistry, but it is currently difficult to achieve. Researchers developed a three-dimensional (3D) cell culture system based on double injectable network (DN) hydrogel for dental pulp regeneration. Due to the kinetic properties of cross-linking, DN hydrogel has ideal injectability under physiological conditions, good mechanical properties, and longer degradation time. The characteristics of DN hydrogel promote the tooth differentiation and mineralization of hDPSCs in vitro [70]. Hydrogel wound dressing shows good application potential in hemostasis, promoting tissue regeneration, epithelial regeneration, and other functions required in wound healing [71,72]. Hydrogel dressing can seal the wound, accelerate hemostasis [73], and reduce the pressure of wound temperature and vasodilation on peripheral nerve fibers [74].

Moreover, hydrogel wound dressing has good deformability and is helpful for wound repair [75]. Due to the ability to cover the postoperative site and maintain the continuous release of drugs, hydrogels even have great potential in preventing tumor recurrence [76]. Through the engineering design of hydrogels, precise control of RNA therapy can be achieved from the hydrogel loading and controlled release of RNA [50]. Furthermore, chitosan is widely used, owning to its biocompatible and degradable nature [77]. Chitosan-based carriers have attracted increasing interest as a safe delivery system for drug, plasmid DNA, and RNA.

DNA double-strand breaks (DS) produced after treatment with ZFN, TALENs, or Cas9 are mainly repaired through three main mechanisms: nonhomologous terminal joining (NHEJ) and HDR [78]. However, during the DNA terminal junction process of NHEJ, insertion or deletion (indel mutation) can be introduced into the DNA sequence of the DSB region. In addition, MMEJ is a variant of NHEJ based on the appearance of microhomology that is classified as highly error-prone [78,79]. The HDR pathway requires donor recombination to restore the correct DNA sequence [80]. This precise modification is desirable for targeted genomic engineering. The paired gRNA in PAM-out orientation mediates HDR efficiently, while the gRNA in PAM-in orientation could hardly mediate HDR [81]. Therefore, the gRNA in PAM-out orientation was employed to mediate HDR. To prevent mutations after repair, synonymous mutations were introduced in the gRNA sequence, that is, the gRNA blockade. The combined use of nCas9 and paired gRNA improves HDR efficiency, but only achieves mild gene repair [82].

It was documented that the base editor ABE could target genomic DNA under the guidance of gRNA, finally achieving the replacement of A•T base pairs to G•C base pairs. For the C175T mutation in PAX9, the advanced ABE, ABE8e, was used to edit A175 on the complementary strand and convert it to C during DNA replication to achieve the repair. Because ABE8e might edit all base A in the gRNA coverage, and T170 and T178 are both in the gRNA coverage of repairing C175T. Theoretically, the corresponding A to T170 and T178 on the complementary strand could be converted to G to varying degrees by ABE8e. That is, T170 and T178 might be converted to C170 and C178, which could be detected by the next deep sequencing. Western blotting was used to find that after modification by ABE8e, the m protein levels of PAX9 were significantly increased, so the conversion of T170C and T178C should not affect the expression of PAX9. Furthermore, the dual luciferase assay showed that the corrected PAX9 possessed elevated transactivation activity. The isolation method of dental pulp stem/progenitor cells from a single human adult colony has been further characterized, demonstrating self-renewal ability, pluripotent differentiation, and in vivo differentiation into odontoblasts to form tubular dentin. Hydroxyapatite/tricalcium phosphate was transplanted into immunocompromised mice [61,83]. Transplantation of human DPSCs into chicken embryos yields a neuronal shape [84]. Transplantation of positive DPSCs into immunosuppressed mice in rhesus monkeys can promote endothelial cell proliferation, neural cell recruitment, and endogenous maturation by increasing ciliary neurotrophic factor signaling to regulate the local microenvironment of neural progenitor cells (CNTF), vascular endothelial growth factor (VEGF), and fibroblast growth factor [85]. These studies have shown that neurogenesis is crucial for dental pulp regeneration. Human adult progenitor cells/stem cells are potential cell-derived diseases for treating nervous system diseases. They can self-renew, have a long proliferative life, and differentiate into neurons, chondrocytes, and adipocytes cultured in vitro [64].

It was reported that DPSCs demonstrated multipotent differentiation capacity into osteogenic, neurogenic, chondrogenic, and adipogenic lineage [86,87]. Therefore, the regulatory effects of hydrogel-delivered ABE8e on the proliferation and differentiation (including osteogenic and neurogenic differentiation) of DPSCs was investigated. Interestingly, hydrogel-ABE8e-modified DPSCs presented higher viability and improved differentiation capacity into osteogenic and neurogenic lineages. In addition, BMP4 promoter is the transcription target of PAX9 [46]. To further validate the efficiency of hydro-gel-delivered ABE8e on C175T correction, the dual-luciferase assay was conducted to test the transactivation ability of PAX9, finding that the modified PAX9 also enhanced transcriptional activation ability.

## 4. Conclusions

In conclusion, the chitosan hydrogel-based nanomedicine was employed to deliver HDR and ABE8e systems to stem cells for the correction of PAX9 mutant. CRISPR/Cas9-based gene editing and stem cells are potential in treating hypodontia induced by genetic mutation.

## 5. Materials and Methods

### 5.1. Preparation and Characterization of Chitosan Hydrogel

The chitosan (Sigma-Aldrich, Burlington, MA, USA) solution (ultrasonic dissolution) was dissolved to a concentration of 1% in 25 mM acetic acid solution under room temperature. Then, the solution was filtered to remove undissolved particles. The pH of the deacetylated chitosan solution was adjusted by adding 1% NaOH solution, stirred well, and then refrigerated for 1–3 h. The obtained solution was dialyzed with 5–8 KD dialysis bag for 1–3 days, and then the hydrogel was centrifuged to remove unconjugated water to prepare the chitosan hydrogel for low-temperature storage. The dried chitosan hydrogel was obtained by drying the hydrogel in a freeze dryer. The chitosan structure of hydrogel was analyzed using a scanning electron microscope (SEM) (ZEISS, Göttingen, Germany). Additionally, the characterization was analyzed using Fourier transform infrared (FTIR) spectroscopy, and the detector was deuterium triglycine sulphate (DTGS) KBr optics. The SEM and FTIR detections were conducted in the Beike 2D materials Co., Ltd. (Suzhou, China).

### 5.2. Cell Culture

Human DPSCs were purchased from Lonza (Catalog #: PT-5025. Basel, Switzerland) and cultured in α-modified Eagle’s medium (α-MEM) (Thermo Fisher Scientific, Waltham, MA, USA). The media were supplemented with 10% fetal bovine serum (FBS, Thermo Fisher Scientific), 100 IU/mL penicillin, and 100 μg/mL streptomycin (Thermo Fisher Scientific). All cells were cultured in a 37 °C humidified atmosphere and 5% CO_2_.

### 5.3. Delivery of DNA by Chitosan Hydrogel

As previously reported [88], chitosan was dissolved in a 25mM acetic acid solution to the concentration of 1%, and the pH was adjusted to 5.5–5.7. Then, both the chitosan solution and 50 mM of sodium sulfate solution containing DNA of 100 mg/mL were heated to 50–55 °C separately. Subsequently, equal volumes of both solutions were quickly mixed under high vertexing for 30 s to obtain nanoparticles. In total, 60–70% confluent DPSCs were incubated with nanoparticles in Opti-MEM, low-serum medium (Thermo Fisher Scientific) for 4 h in a 6-well plate, and then replaced with full growth α-MEM medium.

### 5.4. GFP Expression Plasmid Delivery by Lipofectamine^®^ 3000 or Lentiviral Vector

For the exploration of delivery efficiency, DPSCs were transfected with GFP expression plasmids using Lipofectamine^®^ 3000 transfection reagent (Thermo Fisher Scientific) in 6-well plates. DPSCs were also transduced with lentiviral vector expressing GFP expression in the MOI of 10. 3 days later; the visualization images were taken at GFP channel using a Zeiss microscope (Axio Observer 7, Göttingen, Germany). The fluorescence intensity was monitored in FITC channel using flow cytometry assay (BD FACSCelesta, San Jose, CA, USA).

### 5.5. Generation of the PAX9 Mutant Vector

Total RNA from human DPSCs was extracted using TRIzol reagent (Thermo Fisher Scientific), and the RNA was reverse-transcribed to cDNA using 2 μg of total RNA with PrimeScript^TM^ RT Master Mix (Takara Bio, Shiga, Japan). The wild PAX9 CDS was obtained through PCR, obtaining the 1049bp fragment. The primer sequences of picking PAX9 CDS are as follows: forward, 5′-TGG ATC CGA GCT CGGTAC CC-3′; reverse, 5′-GAG CGC GGA AGC CGT GAC AG-3′. Then, the point mutations C175T was introduced to generate mutated PAX9 CDS sequence through overlap PCR. For overlap PCR, the bridge primers are as follows: forward, 5′-GAT ACA ACG AGA CGG GCT CGA-3′; reverse, 5′-TCG AGC CCG TCT CGT TGT ATC *A*CG CCA GGA TCT TGC-3′. The underlined base in the reverse primer is the part that overlaps with the forward primer; the italic base is for mutation G > A (C > T). The wild (PAX9wild) or mutated (PAX9mut) PAX9 CDS were inserted into the pJET1.2/blunt cloning vector, followed by the insertion in expression vector using digestion–ligation strategy.

### 5.6. Viability Assay

Briefly, DPSCs were seeded at a density of 1 × 104 per well in a 96-well plate and transfected with hydrogel nanoparticles. Cellular viability of DPSCs was detected using the CCK-8 assay kit (Sigma-Aldrich) at 24, 48, 72, 96, and 120 h post transfection. Briefly, CCK-8 assay solution was added to cells in culture and then they were incubated in a cell culture incubator for 2 h at 37 °C. Subsequently, the absorbance values were measured at 450 nm using a microplate reader (BioTek, Shoreline, VT, USA). Triple assays were analyzed.

### 5.7. Generation of Stable Cells

For the in vitro exploration of gene editing, the cells stably carrying PAX9wild or PAX9mut CDS were first generated. PAX9wild or PAX9mut vectors were delivered into DPSCs using the chitosan hydrogel. Three days later, the cells were transferred into ten-centimeter dishes. The selection of stable cells was carried out with puromycin (Thermo Fisher Scientific) at a concentration of 1 μg /mL for 1 week.

### 5.8. Western Blotting Analysis

To detect PAX9 expression with Western blotting, cell lysates were prepared, and the amount of total protein was determined by the BCA Protein Assay Kit (Thermo Fisher Scientific). Twenty micrograms of proteins were separated by ten percent SDS-polyacrylamide gel (SDS-PAGE) and transferred onto a polyvinylidene fluoride (PVDF) membrane (Thermo Fisher Scientific). The blocking of nonspecific binding sites in the membrane was conducted by incubation with 5% nonfat milk for 1 h at room temperature. Subsequently, primary antibody rabbit anti-PAX9 (Thermo Fisher Scientific) and then HRP-conjugated goat antirabbit (Thermo Fisher Scientific) incubation was performed. Tubulin was employed as an internal control. The Chemi-Doc XRS system (Bio-Rad, Hercules, CA, USA) was used to visualize the bands.

### 5.9. Correction of PAX9 Mutant via HDR or ABE8e

For HDR and ABE8e editing, DPSCs were seeded in 24-well plates and transfected at approximately 70% confluency using the Lipofectamine^®^ 3000 transfection reagent. For HDR, the sequences of paired gRNA in PAM-out direction are 5′-CGAGACGGGCTCGATCTTGCC-3′ and 5′-CAGCCGTGCGAGACCCGTAGC-3′; the sequences of paired gRNA in PAM-in direction are 5′-CCGCCAGCTACGGGTCTCGCA-3′ and 5′-CTTGCTGCCCCCGATGGCTCC-3′. For ABE8e editing, the gRNA sequence is 5′-CCGTCTCGTTGTATCACGCCA-3′. For HDR, 1 μg nCas9-double gRNA plasmid and a repair template were transfected per well, the molar ratio for nCas9-double gRNA plasmid to the donor sequence was 1:21. For ABE8e editing, 750 ng of ABE8e plasmid and 250 ng guide of RNA plasmid was transfected per well.

### 5.10. Osteogenic Differentiation

According to published documents [80], osteogenic differentiation was induced. Osteogenic differentiation was performed 72 h post hydrogel delivery of ABE8e. DPSCs were seeded into a 6-well plate at 2 × 103 cells/cm^2^ and cultured in an osteogenic induction medium which consisted of 10% FBS and 10 mM β-glycerolphosphate (Sigma-Aldrich), 10 nM dexamethasone (Sigma-Aldrich), 50 mg/L L-ascorbic acid (Sigma-Aldrich), and 1% penicillin/streptomycin. Then, 21 days later, cells were fixed with 4% paraformaldehyde (PFA) and stained with 2% Alizarin red (Sigma-Aldrich). Finally, the cell pictures were taken by the Zeiss microscope (Axio Observer 7).

### 5.11. Neurogenic Differentiation

To induce neurogenic differentiation, DPSCs at passages 3–5 were seeded into a 6-well plate at 1 × 104 cells/cm^2^ and cultured in a neurogenic induction medium for 7 days. According to reports [80], the induction medium contained DMEM/F12: neurobasal 1:1) supplemented with 0.5% (*v*/*v*) N2, 1% (*v*/*v*) B27 (Thermo Fisher Scientific), 100 μM cyclic adenosine monophosphate (cAMP) (Thermo Fisher Scientific), 20 ng/mL basic fibroblast growth factor (bFGF) (Thermo Fisher Scientific), and 1% penicillin/streptomycin. Finally, the immunofluorescence analysis was performed by Zeiss microscope (Axio Observer 7).

### 5.12. Dual-Luciferase Assay

ABE8e vectors were cotransfected with a BMP4 promoter–reporter construct (p2.8BMP4-Luc), and the phRL-TK plasmid (Promega Corp., San Luis Obispo, CA, USA) was used as the internal control. According to the manuscript, cell extracts were prepared using the Cell Culture Lysis Reagent (Promega Corp.) 72 h post transfection, and the extracts were assayed using the Dual-Luciferase Reporter Assay System (Promega Corp.)

### 5.13. Statistical Analysis

All data were presented as mean ± SD. The statistical significance of the difference between groups was determined by ANOVA or Student’s *t*-test using GraphPad Prism (version 9.2.0, GraphPad, San Diego, CA, USA). *p* < 0.05 was considered statistically significant. All results were from experiments performed in triplicates.

## Data Availability

Not applicable.

## Supplementary Materials

The following supporting information can be downloaded at: https://www.mdpi.com/article/10.3390/gels9060436/s1, Scheme S1: (A) Schematic diagram of PAX9 or PAX9mut expression vector: (B) Western blotting assays for evaluating the expression of PAX9 in DPSCs. NC: DPSCs without treatment. Mock: mock vectors delivered to DPSCs using hydrogel.

## References

[1] Sacks D., Baxter B., Campbell B.C.V., Carpenter J.S., Cognard C., Dippel D., Eesa M., Fischer U., Hausegger K., Hirsch J.A. (2018). Multisociety Consensus Quality Improvement Revised Consensus Statement for Endovascular Therapy of Acute Ischemic Stroke. Int. J. Stroke.

[2] Jowett A.K., Vainio S., Ferguson M.W., Sharpe P.T., Thesleff I. (1993). Epithelial-mesenchymal interactions are required for msx 1 and msx 2 gene expression in the developing murine molar tooth. Development.

[3] Peters H., Neubuser A., Balling R. (1998). Pax genes and organogenesis: Pax9 meets tooth development. Eur. J. Oral Sci..

[4] Yin W., Bian Z. (2015). The Gene Network Underlying Hypodontia. J. Dent. Res..

[5] Bhol C.S., Patil S., Sahu B.B., Patra S.K., Bhutia S.K. (2021). The clinical significance and correlative signaling pathways of paired box gene 9 in development and carcinogenesis. Biochim. Biophys. Acta Rev. Cancer.

[6] Nieminen P., Arte S., Tanner D., Paulin L., Alaluusua S., Thesleff I., Pirinen S. (2001). Identification of a nonsense mutation in the PAX9 gene in molar oligodontia. Eur. J. Hum. Genet..

[7] Zhang H., Gong X., Xu X., Wang X., Sun Y. (2023). Tooth number abnormality: From bench to bedside. Int. J. Oral Sci..

[8] Mattei V., Santacroce C., Tasciotti V., Martellucci S., Santilli F., Manganelli V., Piccoli L., Misasi R., Sorice M., Garofalo T. (2015). Role of lipid rafts in neuronal differentiation of dental pulp-derived stem cells. Exp. Cell Res..

[9] Mattei V., Martellucci S., Pulcini F., Santilli F., Sorice M., Delle Monache S. (2021). Regenerative Potential of DPSCs and Revascularization: Direct, Paracrine or Autocrine Effect?. Stem. Cell Rev. Rep..

[10] Calabrese E.J., Agathokleous E., Dhawan G., Kapoor R., Calabrese V. (2022). Human dental pulp stem cells and hormesis. Ageing Res. Rev..

[11] Lee B.C., Lozano R.J., Dunbar C.E. (2021). Understanding and overcoming adverse consequences of genome editing on hematopoietic stem and progenitor cells. Mol. Ther..

[12] Ding Y., Wang C., Sun Z., Wu Y., You W., Mao Z., Wang W. (2021). Mesenchymal Stem Cells Engineered by Nonviral Vectors: A Powerful Tool in Cancer Gene Therapy. Pharmaceutics.

[13] Sainz-Ramos M., Gallego I., Villate-Beitia I., Zarate J., Maldonado I., Puras G., Pedraz J.L. (2021). How Far Are Non-Viral Vectors to Come of Age and Reach Clinical Translation in Gene Therapy?. Int. J. Mol. Sci..

[14] Qin H., Ji Y., Li G., Xu X., Zhang C., Zhong W., Xu S., Yin Y., Song J. (2022). MicroRNA-29b/graphene oxide-polyethyleneglycol-polyethylenimine complex incorporated within chitosan hydrogel promotes osteogenesis. Front. Chem..

[15] Shepard J.A., Wesson P.J., Wang C.E., Stevans A.C., Holland S.J., Shikanov A., Grzybowski B.A., Shea L.D. (2011). Gene therapy vectors with enhanced transfection based on hydrogels modified with affinity peptides. Biomaterials.

[16] Shafique H., de Vries J., Strauss J., Khorrami Jahromi A., Siavash Moakhar R., Mahshid S. (2023). Advances in the Translation of Electrochemical Hydrogel-Based Sensors. Adv. Healthc. Mater..

[17] Roy A., Manna K., Dey S., Pal S. (2023). Chemical modification of beta-cyclodextrin towards hydrogel formation. Carbohydr. Polym..

[18] Zhu T., Ni Y., Biesold G.M., Cheng Y., Ge M., Li H., Huang J., Lin Z., Lai Y. (2023). Recent advances in conductive hydrogels: Classifications, properties, and applications. Chem. Soc. Rev..

[19] Shen X., Li S., Zhao X., Han J., Chen J., Rao Z., Zhang K., Quan D., Yuan J., Bai Y. (2023). Dual-crosslinked regenerative hydrogel for sutureless long-term repair of corneal defect. Bioact. Mater..

[20] Wang L., Chen G., Fan L., Chen H., Zhao Y., Lu L., Shang L. (2023). Biomimetic Enzyme Cascade Structural Color Hydrogel Microparticles for Diabetic Wound Healing Management. Adv. Sci..

[21] Wang H., Yi X., Liu T., Liu J., Wu Q., Ding Y., Liu Z., Wang Q. (2023). An Integrally Formed Janus Hydrogel for Robust Wet-tissue Adhesive and Anti-postoperative Adhesion. Adv. Mater..

[22] Xu W., Wu Y., Lu H., Zhang X., Zhu Y., Liu S., Zhang Z., Ye J., Yang W. (2023). Injectable hydrogel encapsulated with VEGF-mimetic peptide-loaded nanoliposomes promotes peripheral nerve repair in vivo. Acta Biomater..

[23] Ying Y., Huang Z., Tu Y., Wu Q., Li Z., Zhang Y., Yu H., Zeng A., Huang H., Ye J. (2023). A shear-thinning, ROS-scavenging hydrogel combined with dental pulp stem cells promotes spinal cord repair by inhibiting ferroptosis. Bioact. Mater..

[24] Han X., Tang S., Wang L., Xu X., Yan R., Yan S., Guo Z., Hu K., Yu T., Li M. (2021). Multicellular Spheroids Formation on Hydrogel Enhances Osteogenic/Odontogenic Differentiation of Dental Pulp Stem Cells Under Magnetic Nanoparticles Induction. Int. J. Nanomed..

[25] Amiri H., Aghbashlo M., Sharma M., Gaffey J., Manning L., Moosavi Basri S.M., Kennedy J.F., Gupta V.K., Tabatabaei M. (2022). Chitin and chitosan derived from crustacean waste valorization streams can support food systems and the UN Sustainable Development Goals. Nat. Food.

[26] Rajabi M., McConnell M., Cabral J., Ali M.A. (2021). Chitosan hydrogels in 3D printing for biomedical applications. Carbohydr. Polym..

[27] Kramar R. (1986). The contribution of peroxisomes to lipid metabolism. J. Clin. Chem. Clin. Biochem..

[28] Wang X., Song R., Johnson M., A S., Shen P., Zhang N., Lara-Saez I., Xu Q., Wang W. (2023). Chitosan-Based Hydrogels for Infected Wound Treatment. Macromol. Biosci..

[29] Iacob A.T., Lupascu F.G., Apotrosoaei M., Vasincu I.M., Tauser R.G., Lupascu D., Giusca S.E., Caruntu I.D., Profire L. (2021). Recent Biomedical Approaches for Chitosan Based Materials as Drug Delivery Nanocarriers. Pharmaceutics.

[30] Zhang H., Bahamondez-Canas T.F., Zhang Y., Leal J., Smyth H.D.C. (2018). PEGylated Chitosan for Nonviral Aerosol and Mucosal Delivery of the CRISPR/Cas9 System in Vitro. Mol. Pharm..

[31] Ma L., Shen C.A., Gao L., Li D.W., Shang Y.R., Yin K., Zhao D.X., Cheng W.F., Quan D.Q. (2016). Anti-inflammatory activity of chitosan nanoparticles carrying NF-kappaB/p65 antisense oligonucleotide in RAW264.7 macropghage stimulated by lipopolysaccharide. Colloids Surf. B Biointerfaces.

[32] Doudna J.A., Charpentier E. (2014). Genome editing. The new frontier of genome engineering with CRISPR-Cas9. Science.

[33] Ran F.A., Hsu P.D., Wright J., Agarwala V., Scott D.A., Zhang F. (2013). Genome engineering using the CRISPR-Cas9 system. Nat. Protoc..

[34] Caprifico A.E., Foot P.J.S., Polycarpou E., Calabrese G. (2022). Advances in Chitosan-Based CRISPR/Cas9 Delivery Systems. Pharmaceutics.

[35] Nugrahaningsih D.A.A., Purnomo E., Wasityastuti W., Martien R., Arfian N., Hartatik T. (2022). BMPR2 Editing in Fibroblast NIH3T3 Using CRISPR/Cas9 Affecting BMPR2 MRNA Expression and Proliferation. Indones. Biomed. J..

[36] Srivastav A., Gupta K., Chakraborty D., Dandekar P., Jain R. (2020). Efficiency of Chitosan-Coated PLGA Nanocarriers for Cellular Delivery of SiRNA and CRISPR/Cas9 Complex. J. Pharm. Innov..

[37] Long C., McAnally J.R., Shelton J.M., Mireault A.A., Bassel-Duby R., Olson E.N. (2014). Prevention of muscular dystrophy in mice by CRISPR/Cas9-mediated editing of germline DNA. Science.

[38] Xue H., Wu J., Li S., Rao M.S., Liu Y. (2016). Genetic Modification in Human Pluripotent Stem Cells by Homologous Recombination and CRISPR/Cas9 System. Methods Mol. Biol..

[39] Ran F.A., Hsu P.D., Lin C.Y., Gootenberg J.S., Konermann S., Trevino A.E., Scott D.A., Inoue A., Matoba S., Zhang Y. (2013). Double nicking by RNA-guided CRISPR Cas9 for enhanced genome editing specificity. Cell.

[40] Gaudelli N.M., Komor A.C., Rees H.A., Packer M.S., Badran A.H., Bryson D.I., Liu D.R. (2017). Programmable base editing of A*T to G*C in genomic DNA without DNA cleavage. Nature.

[41] Komor A.C., Kim Y.B., Packer M.S., Zuris J.A., Liu D.R. (2016). Programmable editing of a target base in genomic DNA without double-stranded DNA cleavage. Nature.

[42] Nishida K., Arazoe T., Yachie N., Banno S., Kakimoto M., Tabata M., Mochizuki M., Miyabe A., Araki M., Hara K.Y. (2016). Targeted nucleotide editing using hybrid prokaryotic and vertebrate adaptive immune systems. Science.

[43] Lapinaite A., Knott G.J., Palumbo C.M., Lin-Shiao E., Richter M.F., Zhao K.T., Beal P.A., Liu D.R., Doudna J.A. (2020). DNA capture by a CRISPR-Cas9-guided adenine base editor. Science.

[44] Lawrie G., Keen I., Drew B., Chandler-Temple A., Rintoul L., Fredericks P., Grøndahl L. (2007). Interactions between alginate and chitosan biopolymers characterized using FTIR and XPS. Biomacromolecules.

[45] Rizk A., Rabie B.M. (2013). Electroporation for transfection and differentiation of dental pulp stem cells. Biores. Open Access.

[46] Liang J., Song G., Li Q., Bian Z. (2012). Novel missense mutations in PAX9 causing oligodontia. Arch. Oral Biol..

[47] Holstein M., Mesa-Nunez C., Miskey C., Almarza E., Poletti V., Schmeer M., Grueso E., Ordonez Flores J.C., Kobelt D., Walther W. (2018). Efficient Non-viral Gene Delivery into Human Hematopoietic Stem Cells by Minicircle Sleeping Beauty Transposon Vectors. Mol. Ther..

[48] Putzer B.M., Solanki M., Herchenroder O. (2017). Advances in cancer stem cell targeting: How to strike the evil at its root. Adv. Drug Deliv. Rev..

[49] Kluesner M.G., Lahr W.S., Lonetree C.L., Smeester B.A., Qiu X., Slipek N.J., Claudio Vazquez P.N., Pitzen S.P., Pomeroy E.J., Vignes M.J. (2021). CRISPR-Cas9 cytidine and adenosine base editing of splice-sites mediates highly-efficient disruption of proteins in primary and immortalized cells. Nat. Commun..

[50] Ju Y., Hu Y., Yang P., Xie X., Fang B. (2013). Extracellular vesicle-loaded hydrogels for tissue repair and regeneration. Mater Today Bio..

[51] Schroeder T.B.H., Guha A., Lamoureux A., VanRenterghem G., Sept D., Shtein M., Yang J., Mayer M. (2017). An electric-eel-inspired soft power source from stacked hydrogels. Nature.

[52] Hua M., Wu S., Ma Y., Zhao Y., Chen Z., Frenkel I., Strzalka J., Zhou H., Zhu X., He X. (2021). Strong tough hydrogels via the synergy of freeze-casting and salting out. Nature.

[53] Kamata H., Akagi Y., Kayasuga-Kariya Y., Chung U.I., Sakai T. (2014). “Nonswellable” hydrogel without mechanical hysteresis. Science.

[54] Zhang K., Feng Q., Fang Z., Gu L., Bian L. (2021). Structurally Dynamic Hydrogels for Biomedical Applications: Pursuing a Fine Balance between Macroscopic Stability and Microscopic Dynamics. Chem. Rev..

[55] Salehi S., Naghib S.M., Garshasbi H.R., Ghorbanzadeh S., Zhang W. (2023). Smart stimuli-responsive injectable gels and hydrogels for drug delivery and tissue engineering applications: A review. Front. Bioeng. Biotechnol..

[56] Zhong R., Talebian S., Mendes B.B., Wallace G., Langer R., Conde J., Shi J. (2023). Hydrogels for RNA delivery. Nat. Mater..

[57] Chung H.J., Lee Y., Park T.G. (2023). Thermo-sensitive and biodegradable hydrogels based on stereocomplexed Pluronic multi-block copolymers for controlled protein delivery. J. Control Release.

[58] Vegas A.J., Veiseh O., Doloff J.C., Ma M., Tam H.H., Bratlie K., Li J., Bader A.R., Langan E., Olejnik K. (2016). Corrigendum: Combinatorial hydrogel library enables identification of materials that mitigate the foreign body response in primates. Nat. Biotechnol..

[59] Zhang Z., Gao S., Hu Y.N., Chen X., Cheng C., Fu X.L., Zhang S.S., Wang X.L., Che Y.W., Zhang C. (2022). Ti3 C2 Tx MXene Composite 3D Hydrogel Potentiates mTOR Signaling to Promote the Generation of Functional Hair Cells in Cochlea Organoids. Adv. Sci..

[60] Graziano A., d’Aquino R., Laino G., Papaccio G. (2008). Dental pulp stem cells: A promising tool for bone regeneration. Stem Cell Rev..

[61] Gronthos S., Mankani M., Brahim J., Robey P.G., Shi S. (2000). Postnatal human dental pulp stem cells (DPSCs) in vitro and in vivo. Proc. Natl. Acad. Sci. USA.

[62] Leyendecker Junior A., Gomes Pinheiro C.C., Lazzaretti Fernandes T., Franco Bueno D. (2018). The use of human dental pulp stem cells for in vivo bone tissue engineering: A systematic review. J. Tissue Eng..

[63] Yamada Y., Nakamura-Yamada S., Kusano K., Baba S. (2019). Clinical Potential and Current Progress of Dental Pulp Stem Cells for Various Systemic Diseases in Regenerative Medicine: A Concise Review. Int. J. Mol. Sci..

[64] Nakashima M., Iohara K., Sugiyama M. (2009). Human dental pulp stem cells with highly angiogenic and neurogenic potential for possible use in pulp regeneration. Cytokine Growth Factor Rev..

[65] Dominici M., Le Blanc K., Mueller I., Slaper-Cortenbach I., Marini F., Krause D., Deans R., Keating A., Prockop D., Horwitz E. (2006). Minimal criteria for defining multipotent mesenchymal stromal cells. The International Society for Cellular Therapy position statement. Cytotherapy.

[66] Egusa H., Sonoyama W., Nishimura M., Atsuta I., Akiyama K. (2012). Stem cells in dentistry—Part I: Stem cell sources. J. Prosthodont. Res..

[67] Zayed M., Iohara K., Watanabe H., Ishikawa M., Tominaga M., Nakashima M. (2021). Characterization of stable hypoxia-preconditioned dental pulp stem cells compared with mobilized dental pulp stem cells for application for pulp regenerative therapy. Stem Cell Res. Ther..

[68] Kwack K.H., Lee H.W. (2022). Clinical Potential of Dental Pulp Stem Cells in Pulp Regeneration: Current Endodontic Progress and Future Perspectives. Front. Cell Dev. Biol..

[69] Nakashima M., Akamine A. (2005). The application of tissue engineering to regeneration of pulp and dentin in endodontics. J. Endod..

[70] Han B., Cao C., Wang A., Zhao Y., Jin M., Wang Y., Chen S., Yu M., Yang Z., Qu X. (2023). Injectable Double-Network Hydrogel-Based Three-Dimensional Cell Culture Systems for Regenerating Dental Pulp. ACS Appl. Mater. Interfaces.

[71] Wu J., Pan Z., Zhao Z.Y., Wang M.H., Dong L., Gao H.L., Liu C.Y., Zhou P., Chen L., Shi C.J. (2022). Anti-Swelling, Robust, and Adhesive Extracellular Matrix-Mimicking Hydrogel Used as Intraoral Dressing. Adv. Mater..

[72] Jia B., Li G., Cao E., Luo J., Zhao X., Huang H. (2023). Recent progress of antibacterial hydrogels in wound dressings. Mater Today Bio..

[73] Jiang Y., Wang J., Zhang H., Chen G., Zhao Y. (2022). Bio-inspired natural platelet hydrogels for wound healing. Sci. Bull..

[74] Yun S., Greco V. (2022). From start to finish—A molecular link in wound repair. Science.

[75] Bertsch P., Diba M., Mooney D.J., Leeuwenburgh S.C.G. (2023). Self-Healing Injectable Hydrogels for Tissue Regeneration. Chem. Rev..

[76] Qian Q., Song J., Chen C., Pu Q., Liu X., Wang H. (2023). Recent advances in hydrogels for preventing tumor recurrence. Biomater. Sci..

[77] Desai N., Rana D., Salave S., Gupta R., Patel P., Karunakaran B., Sharma A., Giri J., Benival D., Kommineni N. (2023). Chitosan: A Potential Biopolymer in Drug Delivery and Biomedical Applications. Pharmaceutics.

[78] Haber J.E. (2000). Partners and pathwaysrepairing a double-strand break. Trends Genet..

[79] Smirnikhina S.A., Zaynitdinova M.I., Sergeeva V.A., Lavrov A.V. (2022). Improving Homology-Directed Repair in Genome Editing Experiments by Influencing the Cell Cycle. Int. J. Mol. Sci..

[80] Thompson L.H., Schild D. (2001). Homologous recombinational repair of DNA ensures mammalian chromosome stability. Mutat. Res..

[81] Saifaldeen M., Al-Ansari D.E., Ramotar D., Aouida M. (2020). CRISPR FokI Dead Cas9 System: Principles and Applications in Genome Engineering. Cells.

[82] Wang Y., Zhao J., Duan N., Liu W., Zhang Y., Zhou M., Hu Z., Feng M., Liu X., Wu L. (2018). Paired CRISPR/Cas9 Nickases Mediate Efficient Site-Specific Integration of F9 into rDNA Locus of Mouse ESCs. Int. J. Mol. Sci..

[83] Gronthos S., Brahim J., Li W., Fisher L.W., Cherman N., Boyde A., DenBesten P., Robey P.G., Shi S. (2002). Stem cell properties of human dental pulp stem cells. J. Dent. Res..

[84] Arthur A., Rychkov G., Shi S., Koblar S.A., Gronthos S. (2008). Adult human dental pulp stem cells differentiate toward functionally active neurons under appropriate environmental cues. Stem Cells.

[85] Huang A.H., Snyder B.R., Cheng P.H., Chan A.W. (2008). Putative dental pulp-derived stem/stromal cells promote proliferation and differentiation of endogenous neural cells in the hippocampus of mice. Stem Cells.

[86] Zhang Y., Liu J., Zou T., Qi Y., Yi B., Dissanayaka W.L., Zhang C. (2021). DPSCs treated by TGF-beta1 regulate angiogenic sprouting of three-dimensionally co-cultured HUVECs and DPSCs through VEGF-Ang-Tie2 signaling. Stem Cell Res. Ther..

[87] Jia Q., Chen X., Jiang W., Wang W., Guo B., Ni L. (2016). The Regulatory Effects of Long Noncoding RNA-ANCR on Dental Tissue-Derived Stem Cells. Stem Cells Int..

[88] Kiang T., Wen J., Lim H.W., Leong K.W. (2004). The effect of the degree of chitosan deacetylation on the efficiency of gene transfection. Biomaterials.

