# Supplementary material for: Chitosan Hydrogel-Delivered ABE8e Corrects PAX9 Mutant in Dental Pulp Stem Cells
Source: Gels. 2023 May 25;9(6):436. doi: 10.3390/gels9060436 (PMC10297230; doi:10.3390/gels9060436)
Supplement: Supplementary file 1 [file gels-09-00436-s001.zip › gels-2335494-supplementary.pdf]

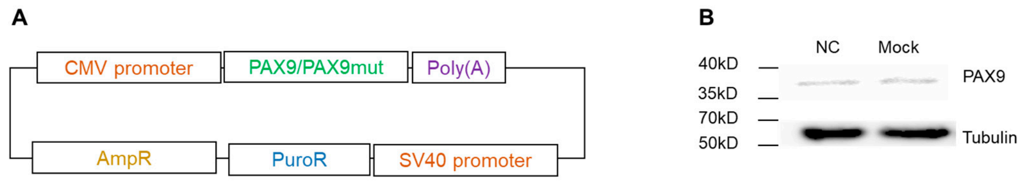

Scheme S1. (**A**) Schematic diagram of PAX9 or PAX9mut expression vector: (**B**) Western blotting assays for evaluating the expression of PAX9 in DPSCs. NC: DPSCs without treatment. Mock: mock vectors delivered to DPSCs using hydrogel.
